# Traces of a prehistoric and potentially tsunamigenic mass movement in the sediments of Lake Thun (Switzerland)

**DOI:** 10.1186/s00015-022-00405-0

**Published:** 2022-04-09

**Authors:** Katrina Kremer, Stefano C. Fabbri, Frederic M. Evers, Nora Schweizer, Stefanie B. Wirth

**Affiliations:** 1 Swiss Seismological Service, ETH Zürich, Sonneggstrasse 5, 8092 Zurich, Switzerland; 2 Insitute of Geological Sciences and Oeschger Centre for Climate Change Research, Baltzerstrasse 1+3, University of Bern, 3012 Bern, Switzerland; 3 Laboratory of Hydraulics, Hydrology and Glaciology (VAW), ETH Zurich, 8093 Zurich, Switzerland; 4 Centre for Hydrogeology and Geothermics (CHYN), University of Neuchatel, Emile-Argand 11, 2000 Neuchatel, Switzerland; 5 GEOTEST AG, Bernstrasse 165, 3052 Zollikofen, Switzerland

**Keywords:** Mass movement, Prehistoric, Lake sediments, Lake Thun, Impulse wave, Lacustrine tsunami

## Abstract

Mass movements constitute major natural hazards in the Alpine realm. When triggered on slopes adjacent to lakes, these mass movements can generate tsunami-like waves that may cause additional damage along the shore. For hazard assessment, knowledge about the occurrence, the trigger and the geomechanical and hydrogeological mechanisms of these mass movements is necessary. For reconstructing mass movements that occurred in or adjacent to lakes, the lakes’s sedimentary record can be used as an archive. Here, we present a prehistorical mass-movement event, of which the traces were found in an alpine lake, Lake Thun, in central Switzerland. The mass movement is identified by large blocks on the bathymetric map, a chaotic to transparent facies on the reflection seismic profiles, and by a mixture of deformed lake sediments and sandy organic-rich layers in the sediment-core record. The event is dated at 2642–2407 cal year BP. With an estimated volume of ~ 20 × 10^6^ m^3^ it might have generated a wave with an initial amplitude of > 30 m. In addition to this prehistorical event, two younger deposits were identified in the sedimentary record. One could be dated at 1523–1361 cal year BP and thus can be potentially related to an event in 598/599 AD documented in historical reports. The youngest deposit is dated at 304–151 cal year BP (1646–1799 AD) and is interpreted to be related to the artificial Kander river deviation into Lake Thun (1714 AD).

**Supplementary Information:**

The online version contains supplementary material available at 10.1186/s00015-022-00405-0.

## Introduction

In the Alps, mass movements represent a major natural hazard, especially within the framework of a changing climate. Causes of such mass movements can be earthquakes, climate-driven processes including heavy rainfall, permafrost thawing and ice melt, or human activity (e.g., quarries).

One of the most recent example in Switzerland is the failure of a rock volume of about 3 × 10^6^ m^3^ on Pizzo Cengalo in 2017, which developed into a debris flow and led to casualties and damage to infrastructure in the valley (Walter et al., 2020). Other examples from Switzerland are the landslide of Randa in 1991 mobilising around 30 × 10^6^ m^3^ of rock that fell down the Matter Valley impounding a landslide-dammed lake, and the rockfall of Fidaz in 1939 with a volume of 100,000 m^3^ that caused 18 casualties and substantial damages in the commune of Flims. The area of Flims is also known for the largest landslide that occurred in the Alps with a volume of ~ 8–10 × 10^9^ m^3^ (Abele, 1974; Heim, 1931; Poschinger et al., 2006). It is dated at 9660–9430 cal year BP based on lake sediments (Deplazes et al., 2007).

Lake sediments are excellent archives of past mass movements (e.g., rockfalls, rock avalanches and landslides) when they occurred on the slopes adjacent to a lake, as the mobilized sediment/rock masses are deposited in the lake basins. Due to continuous sedimentation in the lake such mass-movement deposits are preserved in the lacustrine record. Examples of deposits of prehistorical subaerial mass movements recorded in Swiss lakes are those in Lake Lucerne (Schnellmann et al., 2006) and in Lake Oeschinen (Knapp et al., 2018). Some Austrian examples of prehistorical landslides were found in Lake Mondsee (Daxer et al., 2018) and in Lakes Plansee and Piburgersee (Oswald et al., 2021) and the age of the Monte Peron landslide in nothern Italy has recently been revised using lake sediments (Zolitschka et al., 2021). More examples of subarerial mass movements recorded in lakes exist (e.g., Chile; Van Daele et al., 2015; US; Leithold et al., 2019 amongst others).

Besides representing a natural hazard by themselves, mass movements can generate tsunami-like waves—so-called impulse waves—when propagating into a lake or any other type of water reservoir, thereby causing through cascading effects even more damage than the landslide itself. A recent example of a tsunami that was caused by a terrestrial landslide occurred in Lake Chehalis, Canada, in 2007 (Roberts et al., 2013). Further examples of terrestrial landslides into lakes that triggered an impulse wave are Lake Loen, Norway, in 1905 and 1936 (Grimstad & Nesdal, 1991), Lago Cabrera, Chile, in 1960 (Watt et al., 2009), Yanahuin Lake, Peru, in 1971 (Plafker & Eyzaguirre, 1979), Lake Askja, Iceland, in 2014 (Gylfadottir et al. 2017), and Lakes Calafquén, Pellaifa and Rupanco, Chile, in 1960 (Van Daele et al., 2015). Some historical examples from Switzerland include: (1) the 1601 AD Unterwalden tsunami on Lake Lucerne that was caused by earthquake-triggered terrestrial and subaquatic mass movements (Keller, 2019; Schnellmann et al., 2002), (2) the 1801 AD rockfall in Sisikon that also triggered an impulse wave in Lake Lucerne that caused more than 10 fatalities (Huber, 1982), (3) the 1963 and 1964 Obermatt quarry rockfalls (with 20 × 10^3^ m^3^ and 70 × 10^3^ m^3^, respectively; Huber, 1982) that generated a near-source wave run-up of 4 and 15 m, respectively at the quarry and of several meters on the opposite side of Lake Lucerne (3.5 km away from the source) for the 1964 event. The Goldau Landslide in 1806 AD generated soft sediment mobilization in the swampy plain that in turn generated a 15 m high wave on Lake Lauerz (Bussmann & Anselmetti, 2010). Further examples of historical and prehistorical landslide-induced impulse waves that are known to have occurred in Swiss lakes and world-wide are given in e.g., Huber, (1982); Heller, (2009); Roberts et al. (2014) and Kremer et al. (2021). Only few examples of prehistorical mass-movement-generated impulse waves are reported, as it is difficult to identify prehistorical tsunami deposits within lacustrine environments (Kremer et al., 2021). Another way to infer the occurrence of prehistorical mass-movement-induced impulse waves in lakes is to model the tsunamigenic potential. These models may be based on generally applicable equations from hydraulic laboratory tests or hydrodynamic numerical simulations (Evers et al., 2019; Yavari-Ramshe & Ataie-Ashtiani, 2017). In both cases, the slide characteristics, e.g., slide impact velocity or slide volume, represent the governing input parameters.

In Lake Thun, located in central Switzerland, traces of a mass movement were found in the sedimentary record and were previously attributed to a historic event in 598/599 AD (Wirth et al., 2011). This event was documented by the chronist Fredegarius who mentions in his reports of the years 598/599, a “boiling lake” and “dead fishes washed ashore” (Fredegarius, 1888). The observation of the lake’s agitation may describe the consequence of an impulse wave that was triggered by a mass movement into the lake. In order to better understand the chronist’s observations and to verify the hypothesis of a lake tsunami, we combined geophysical imaging of the lake floor and the sedimentary record, sediment cores up to 11 m-long and impulse-wave modelling. Thus in this study, (1) we present multiple lines of evidence of deposits related to terrestrial mass-movement events based on multibeam bathymetry, reflection seismic profiles and sediment-core data, (2) we date the mass-movement deposits, and (3) we investigate if the largest event was potentially tsunamigenic.

In this study, we use the generic term ‘mass movement’ when the specific mechanism of the process is unknown.

## Regional setting

Lake Thun is located in central Switzerland, at the northern front of the Swiss Alps. The northern shore lies from East to West in the tectonic units of the Subalpine Molasse, the Subalpine Flysch Zone and the Helvetic nappes while the southern shore is located in the Peninic (West) and Helvetic (East) units (Swisstopo, Geologischer Atlas 1:25,000, Blatt Beatenberg in preparation; Swisstopo 2008 Tektonische Karte der Schweiz 1:500,000 Bundesamt für Landestopographie). The valley of Lake Thun was formed through glacial erosion of the Aare glacier during the last glaciations. The overdeepening was likely caused by a combination of subglacial erosional processes and geologic predispositions related to lithologic (erosion-sensitive units) and tectonic controlling factors (e.g., Hohgant-Sundlauenen fault; Fabbri et al., 2018). While the northeastern shore of the lake is dominated by steep slopes without major glacial deposits the southwestern shore is characterised by a more gently dipping topography and several shore-parallel moraine ridges (Fabbri et al., 2017).

Lake Thun is a freshwater lake with a water volume of around 6.42 km^3^ and a maximum water depth of 216.4 m (with respect to the 75-year-long average water level of 557.7 m a.s.l.). The main tributaries of the lake are the Kander and the Aare River. While the Aare provides 50% of the water supply but is almost free of sediment particles as it flows out of upstream Lake Brienz, the Kander River was deviated in 1714 AD into Lake Thun and provides nowadays 40% of the water supply and 85% of the sediment supply (Sturm & Matter, 1972; Wirth et al., 2011). Its deviation into the lake thus drastically increased the sediment input.

**Fig. 1:**
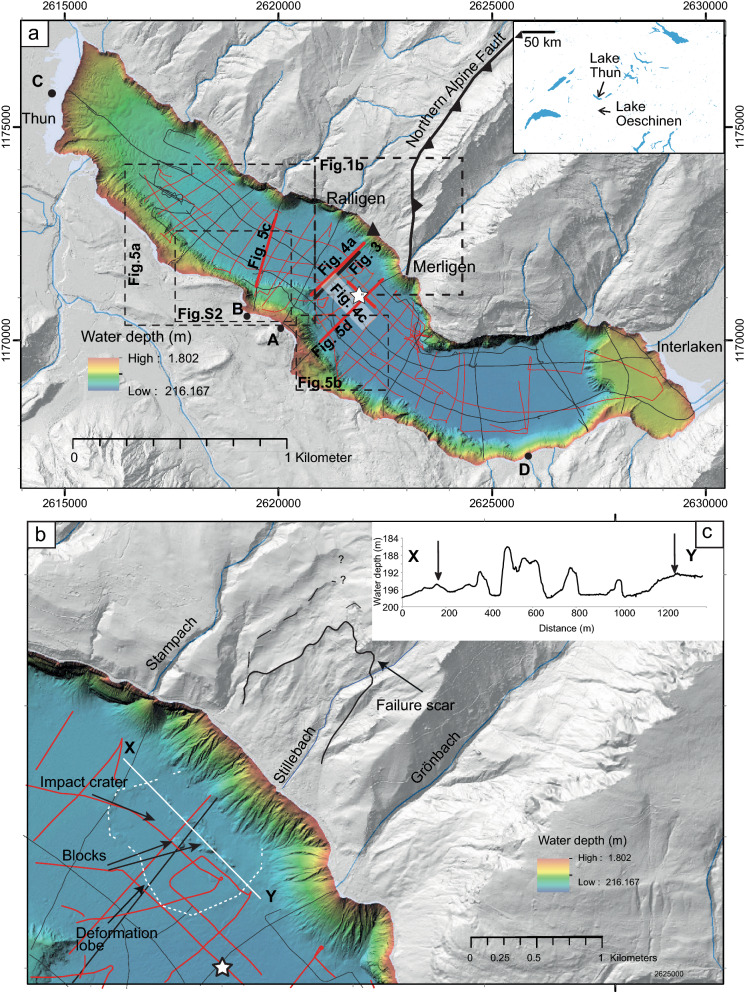
Hillshade of the bathymetric map of Lake Thun and the sourrounding area. **a** Map of the study area showing the location of the airgun (black lines) and the pinger (red lines) reflection seismic lines and the position of the long sediment core (white star). Bold lines represent the reflection seismic profiles that are shown in Figs. 3, 4 and 5. The black triangle shows the slide impact location for the simulation of the impulse wave. A, B, C and D refer to the locations where wave run-up has been estimated. **b** Enlargement of the detachment area on land and the depositional area of the mass movement in the lake. The dashed white line marks the the deformation lobe (“amphitheatre”). **c** Lake bottom profile of the X–Y transect (position is shown with a white line in **b**). Source of the terrestrial digital elevation model: Swisstopo, Federal Office of Topography

The bathymetric map of Lake Thun shows, on a northwest to east axis, a basin with a smoothly dipping slope (0.5–1°) from the Kander delta towards the southeast until reaching the deep basin with the maximum water depth. The eastern part of the lake, where the Aare River enters, is characterized by a platform with a water depth of around 80 m. The on-shore topography, with the steep northern and the flatter southern shore, can be extrapolated to the submerged slopes. The submerged northern slopes are generally steep (up to 80°) and the distance shore-to-slope-toe shorter than 100 m. Exceptions are delta fans and the area of interest of this study that is characterized by slope gradients of 40° and a distance shore-to-slope-toe of up to 300 m (Fig. 1a and Additional file 1: Fig. S1). The southern sublacustrine slope is in contrast characterized by a morphological step at Spiez (between points A and B on Fig. 1a) and the occurrence of mass movements (as shown in Fabbri et al., 2017 and Additional file 1: Fig. S2). The bedrock is covered by up to 500 m of sediments in the deeper part of the basin as shown from seismic reflection data (Fabbri et al., 2018; Matter et al., 1971).

## Methods

### Multibeam bathymetry

High-resolution bathymetric data acquisition was performed by Fabbri et al. (2017) in 2014 using a Kongsberg EM2040 multibeam echosounder (Kongsberg Maritime, Horten, Norway) in 1° by 1° beam-width configuration, operating at 300 kHz (Fig. 1). The positioning was performed with a Leica GX1230 GNSS receiver (Leica Geosystems, Heerbrugg, Switzerland) in combination with the swipos GIS/GEO real-time kinematic positioning (Swiss Federal Office of Topography, Swisstopo, Wabern, Switzerland). The survey lines were recorded in a shore-parallel pattern. During processing, all auxiliary sensor data (motion sensor, heading sensor, sound velocity sensor, positioning) are merged, reviewed and manually corrected if necessary. Resulting point clouds were reviewed and different algorithms for rasterizations of the point clouds were tested. For analysis, a gridded data set with a 1 m-cell size was produced.

### Reflection seismic survey

Several reflection seismic campaigns have been conducted on Lake Thun.

In 2007, (Wirth et al., 2011) acquired single-channel reflection seismic profiles with a 3.5 kHz pinger system. In 2017, additional pinger reflection seismic profiles were acquired in the framework of this study. For both acquisition campaigns, positioning was performed with a Garmin GPS with an accuracy of 2–5 m. The seismic data have a theoretical vertical resolution of 0.1 m. Further processing steps included band-pass filtering with seismic processing workshop software SPW (Parallel Geoscience Corporation).

In 2015, 180 km of multi-channel airgun reflection seismic profiles were recorded with a Sercel two-chamber Mini GI airgun (15/15 in^3^) combined with a Geometrics MicroEl streamer of 97 m length and 24 channels (Fabbri et al., 2017). The positioning of the vessel was measured with a Garmin GPSmap 76Cx GPS with an accuracy of 2–5 m. The dominant main frequency was 140 Hz giving a theoretical vertical resolution of 2.5 m at a sound velocity of 1500 m/s. Further processing steps included: frequency band pass filtering, muting, velocity-model creation based on normal-move-out (NMO) analysis and recorded sound-velocity profiles from bathymetric surveys, multiple suppression using surface-related multiple elimination, NMO corrections and CMP stacking, post-stack FX-deconvolution, and post-stack Kirchhoff depth migration.

The interpretation of the reflection seismic data was performed with Kingdom Suite 2015.

### Sediment coring and analysis

Using the reflection seismic data for choosing the best position, an 11 m-long sediment core (THU18-Ku-03) was retrieved from a water depth of around 200 m (Fig. 1b, white star). The coring system used was a modified Kullenberg-type gravity piston coring system (Kelts et al., 1986). In the laboratory, the core was split in halves, photographed and the sediments were visually described. Samples of terrestrial organic remains were taken to be dated by accelerator mass spectrometry (AMS) ^14^C dating.

### Age-depth model

The age-depth model is based on nine AMS ^14^C dates (Table 1) from leave remains. In addition to the ^14^C dates, further historical events were used as time markers in the age-depth model (Table 1). The ^137^Cs Peaks of 1963 (peak of nuclear weapon testing) and 1986 (Chernobyl nuclear accident) were used as historical event points in the age model. The depth of these horizons was defined by core correlation with older sediment cores from the study of Wirth et al. (2011). Similarly, the 1852 and 2005 flood events were used as historical marker horizons. The core-to-core correlation with the dated older cores of the Wirth et al. (2011) study is shown (Additional file 1: Fig. S3). For the age-depth modeling, we corrected the total core depth for near-instantaneous deposits by removing event layers > 2 cm (Additional file 1: Table S1) and used this new event-deposit-corrected depth.

**Table 1:** Key elements for age depth-model

Core section	Sample number	Sample depth (cm)	Corrected sample depth (cm)	Sample age (^14^C year BP)	Sample age (AD)	Sample age (cal year BP) 2 sigma interval	Dated material
THU18-Ku-3SC_4	Surface	0	0	− 68	2018		
THU18-Ku-3SC_10	Hist. Flood	6.6	6.6	− 55	2005		
THU18-Ku-3SC-24	Cs Peak	20	19.1	− 36	1986		
THU18-Ku-3SC-40	Cs Peak	36	33.1	− 13	1963		
THU18-Ku-3C-68	Hist. Flood	134	121.4	98	1852		
THU18-Ku-3E-61	ETH-94965	235.1	169.8	354 ± 26		405.5 ± 89.5	Leaf remains
THU18-Ku-3G-42	ETH-94958	413.4	337.8	1404 ± 26		1316.5 ± 29.5	Leaf remains
THU18-Ku-3I-57	ETH-94957	531.3	426.8	1590 ± 22		1473 ± 60	Leaf remains
THU18-Ku-3I-91	ETH-94956	565.3	458.7	1663 ± 22		1571 ± 41	Leaf remains
THU18-Ku-3J-78	ETH-94959	645	536.3	1924 ± 28		1874.5 ± 56.5	Leaf remains
THU18-Ku-3K-34.5	ETH-94950	699.2	583.6	2204 ± 28		2230.5 ± 82.5	Leaf remains
THU18-Ku-3-K-92	ETH-105349	756.7	640.6	2318 ± 22		2336.5 ± 17.5	Leaf remains
THU18-Ku-3L-19.5	ETH-94948	784.7	669.1	2452 ± 34		2532.5 ± 171.5	Leaf remains
THU18-Ku-3N-80	ETH-94960	1047.1	761	2977 ± 23		3142.5 ± 70.5	Leaf remains

Summary of dating horizons such as historical flood events, ^137^Cs peaks and radiocarbon dates from leaf remains used for the age model of sediment core THU18-Ku-03 (position in Fig. 1b). The total core depth and the event-deposit-corrected depth is given. The core-to-core correlation that illustrates the position of the flood events and the ^137^Cs peaks is shown in Additional file 1: Fig. S3

For the calibration of the ^14^C dates and the generation of the age-depth model, we used the package ‘rbacon 2.5.7’ (Blaauw & Christen, 2011, https://chrono.qub.ac.uk/blaauw/bacon.html) for the open-source statistical software’ R’. The calibration command uses the Northern Hemisphere terrestrial IntCal13 calibration curve (Reimer et al., 2013). ‘rbacon’ produces an age-depth model based on bayesian statistics. The age-depth model allows extracting ages for selected depths.

### Volume calculations

The off- and on-shore volumes of the mass movement were calculated with ArcGIS.

For the off-shore volume of the mass-movement deposit, the top and the bottom of the deposit were defined on the reflection seismic profils using Kingdom Suite. The two-way travel times of these horizons were then imported into ArcGIS and converted into depth using a constant velocity of 1500 ms^−1^. A thickness map was created and finally the volume of the deposit was calculated.

**Fig. 2:**
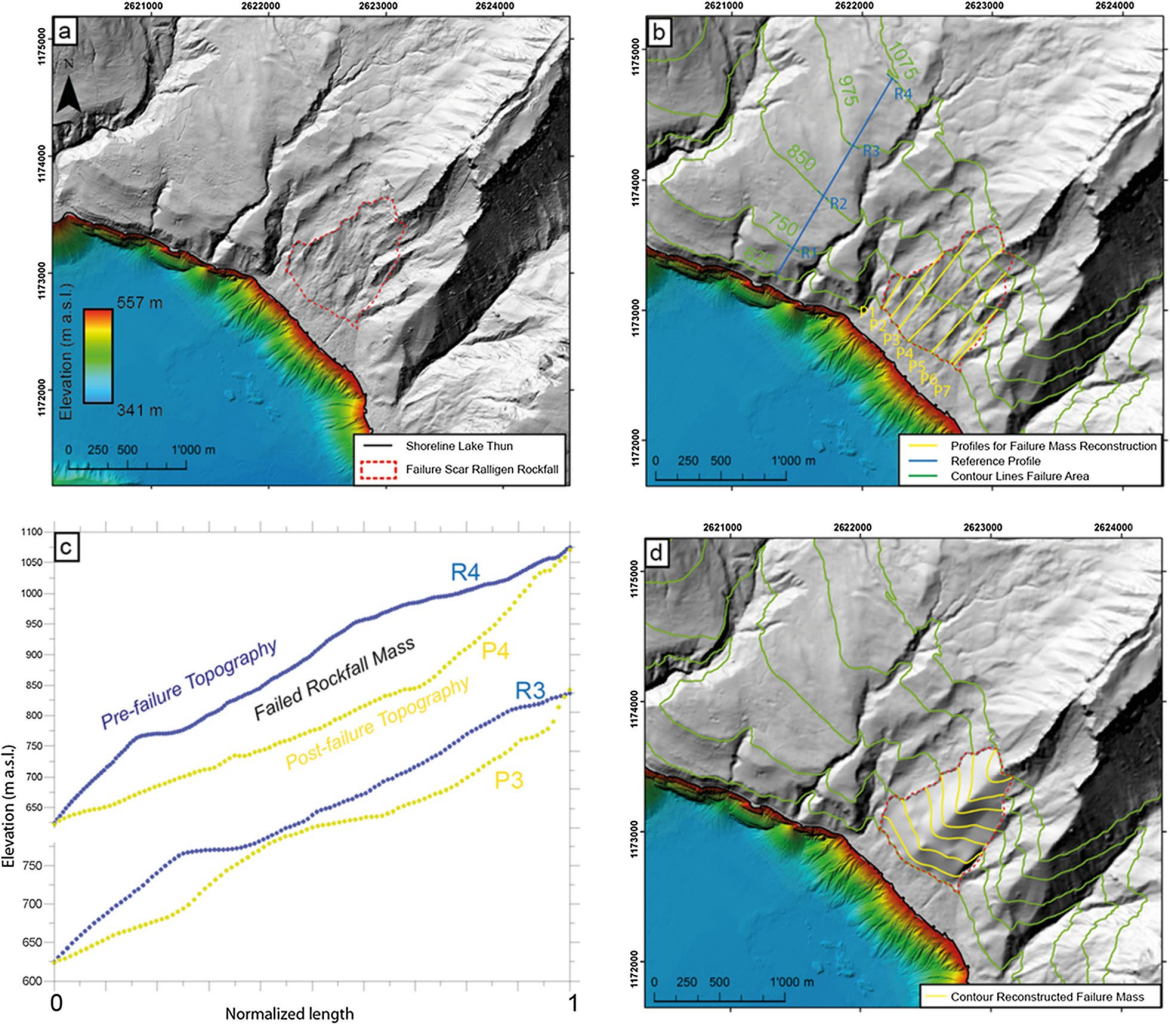
Pre-failure topography reconstruction on-shore. **a** Digital elevation model (SwissAlti3D Swisstopo, Swiss Federal Office of Topography, 315/45 illuminated) with bathymetric map superimposed. Failure scar of the Ralligen Rockfall is illustrated. **b** Choosing an intact reference profile (blue) nearby the failed area and projecting it on to the failure zone along profiles P1-P7. **c** Comparison between post-failure and pre-failure topography for two selected profiles at P3 and P4. **d** Reconstructed topography with contour lines based on 7 projected profiles

For the estimation of the failed volume on shore, the failure scar of the potentially largest event was first defined (Fig. 2a) and the selected elevation contour lines were displayed. The “freshest” scar, determined by the surface roughness, was considered for the pre-failure topography reconstruction. The bottom of the failure area does not show a clear failure scar, but is rather blurred due to the smooth transition from failure to depositional area. Therefore, the lower limit of the failure area was assumed to be approximately horizontal. The pre-failure topography was reconstructed using a reference profile (blue line R in Fig. 2b) close to the failure area, ideally mimicking the intact topography (Fig. 2b). The reference profile was then projected onto a set of 7 profiles (P1–P7) across the failure area. The length of the reference profiles (R1-R4, blue lines in Fig. 2b) was adjusted to meet the elevation contour at the starting and end point of the projected profiles P1 to P7 (e.g., normalization of length R3 until 975 m asl for profiles P3 and P5, and of length R4 until 1075 m asl for profile P4, Fig. 2b, c). This procedure ensures that the resulting pre-failure topography incorporates prominent morphologic features, such as a mountain crest (Fig. 2d). A thickness map was built based on the difference between the reconstructed pre-failure and today’s topography and the failed volume was calculated.

### Impulse wave assessment

In order to determine if the mass movement had been tsunamigenic, the computational procedure proposed by Evers et al. (2019) was applied. The procedure combines generally applicable equations derived from hydraulic laboratory experiments and includes the following three stages of a landslide tsunami event: wave generation, wave propagation and run-up. As the investigated mass movement is interpreted as a terrestrial one, the tsunami-like waves are specifically referred to as impulse wave.

The slide impact velocity $V_{s}$, as one of the most important governing parameters, was estimated considering the energy balance of a frictional block slide model on an inclined plane (Kesseler et al., 2020; Körner, 1977) as

1 $$ V_{s} = \sqrt {2g\Delta z_{sc} \left( {1 - \tan \delta \cot \alpha } \right)}$$

with gravitational acceleration *g*, drop height of the slide centroid Δ*z*_*sc*_, dynamic bed friction angle *δ*, and slope angle *α*. Additional governing parameters for the wave generation are the bulk slide volume $\rlap{--} V_{s}$ (here we used the mass-movement volume calculated based on the seismic reflection data; see Results), slide thickness *s*, slide width *b*, bulk slide density *ρ*_*s*_, and still water depth *h*. Four wave propagation profiles originating at the slide impact location (triangle in Fig. 1A) were selected: Bürg (A—hill on the direct opposite shore), Spiez (B—town), Thun (C—town), Leissigen (D—municipality). The locations are shown in Fig. 1. In addition to the general run-up equation by Evers and Boes (2019) included in the computational procedure by Evers et al. (2019), run-up heights were also computed following the approach by Kastinger et al. (2020) which considers the wave’s position within the generated wave train.

## Results and interpretation

### Traces of a mass movement in the digital bathymetric and elevation models

In the central part of Lake Thun, at around 1 km from the NE shore between the river deltas of Stampach and Grönbach, the digital bathymetric model (DBM) of the lake floor shows blocky shaped features within an “amphitheater” morphology that stand out from the surrounding flat basin (Fig. 1b). This amphitheater morphology (white dashed line on Fig. 1b) is delimited by a rim 2–5 m higher in elevation compared to the inner amphitheater (Fig. 1c). The large blocks (hummocky structure) reach sizes up to 35 m × 25 m × 10 m (arrows in Fig. 1c). This amphitheatre structure in combination with the blocks on the lake floor is considered as the trace of a mass movement. The amphitheatre structure is also called “halo” structure by Hilbe and Anselmetti (2014) and is interpreted as the deformation front in the underlying sediments (Hilbe and Anselmetti, 2014), similar to subaquatic mass movements (Sammartini et al., 2021). Similar features are observed by terrestrial mass movements in fjord or other lakes (e.g., Hilbe et al., 2011; Van Daele et al., 2013). Within the DBM no traces of a potential failure scar are visible on the northeastern sublacustrine slope, suggesting a terrestrial origin of the mass movement. However, at the location of the mass movement the submerged slope is gentler (> 30–40°) than the neighbouring slopes (not influenced by delta fans of inflows) where the slope gradient is up to 80° (Additional file 1: Fig. S1). As the terrestrial terrain is characterized by only small inflows (Stillebach River and another smaller one), the gentler slope angle at the studied location is interpreted to be the result of the deposition of a terrestrial mass movement.

Indeed, on the digital elevation model (DEM), on-shore traces such as a potential failure scar on the mountain’s slopes on the nothern shore are present, especially between Ralligen and Merligen (Fig. 1). Herb et al. (1978) described for this area debris of a mass movement between the fort of Ralligen and Merligen. On the DEM, several failure scars are visible, as highlighted on Fig. 1b. The failure scar and structure with the roughest surface is interpreted as the largest and most recent failure. On the slope the failure area is > 100 m long (e.g., profile P3/P4 on Fig. 2) and it is located in the Subalpine Flysch Zone consisting of marl, marly limestones and sandstone. The failed area covers a surface of around 1000 × 700 m and is recognized based on a rougher topography compared to neighbouring slope topography. This area is delimited by a continuous scar on the western side while the eastern side is less defined and could also include potentially partly the Stillbach canyon (Fig. 1b).

### Reflection seismic data

**Fig. 3:**
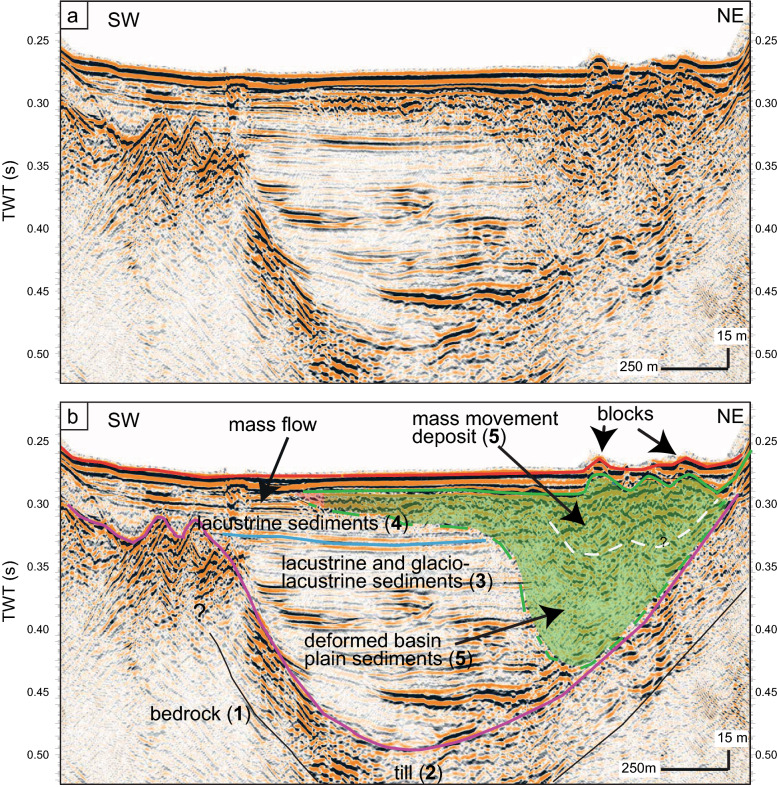
Uninterpreted (**b**) and interpreted (**b**) SW-NE airgun reflection seismic profile (the geological units have been interpreted by Fabbri et al., 2017) (for position of profile see Fig. 1a). The numbers in** a** refer to the seismic facies described in the text. The inherited positive relief is interpreted as the rocky blocks seen in the bathymetry (Figs. 1 and 2). The facies marked in green represents the interpreted volume affected by the event. The lower, more transparent facies is interpreted as the deformed basin plain sediments due to the impact of the rock mass while the upper chaotic facies with high-amplitude discontinuous reflections is interpreted as the failed rock mass

Several reflection seismic profiles cross the area that can be linked to the mass-movement impact (Fig. 1). Figure 3 shows a Southwest to Northeast oriented airgun reflection seismic profile that crosses the deposit (position on Fig. 1). This profile that has been previously interpreted by Fabbri et al. (2018), shows from bottom to top different seismic facies: (1) laterally continuous and low-amplitude reflections that are interpreted as bedrock, (2) high-amplitude, discontinuous, lateral and partly chaotic reflections interpreted as glacial till, (3) horizontally layered, partly low-amplitude reflection interpreted as glacio-lacustrine and lacustrine deposits of the Late Glacial and (4) high-amplitude, horizontal, parallel and continuous reflections interpreted as Holocene sediments. The general geometry of these units and thickness distribution have been previously presented and discussed in Matter et al. (1971) and Fabbri et al., (2017, 2018) and will not be further discussed in this study. The glacio-lacustrine and lacustrine sediments of the Late Glacial and the Holocene sediments are disturbed by (5) a transparent to chaotic facies with some high-amplitude discontinuous reflections that can be partly followed up to the sediment surface. This chaotic seismic facies is interpreted as being associated to a mass-movement deposit and most probably linked to the mass-movement traces imaged in the bathymetric dataset. While the lower, more transparent part of this facies is interpreted as deformed basin plain sediments caused by the impact of the rock mass, the upper part constituted of high-amplitude discontinuous and chaotic reflections is interpreted to partly represent the rock mass itself. A clear distinction between rock mass and deformed basin plain sediments cannot be made and a basal shear surface cannot be identified (in analogy to Van Daele et al., 2013 for example) in the airgun data. In the most proximal part (northeastern shore) the entire sediment sequence overlying the till deposits [seismic facies (2)] is deformed.

**Fig. 4:**
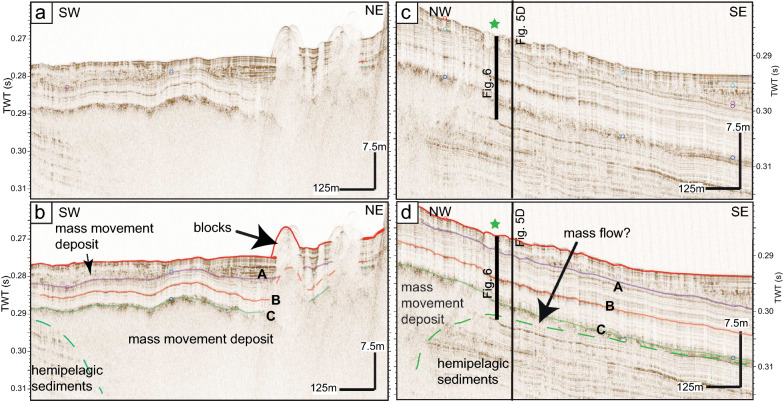
Uninterpreted (**a** and **c**) and interpreted (**b** and **d**) high-resolution 3.5 kHz reflection seismic profiles. Profile **a**/**b** is a SW-NE profile crossing the discussed mass-movement deposit. Profile **c**/**d** is a NW–SE profile imaging the seismic facies at the coring position (marked with a star on the profile and Fig. 1), the black rectangle indicates the penetration depth of the sediment core. The vertical black line shows the intersection with the profile in Fig. 5d. Profile locations are shown in Fig. 1a

**Fig. 5:**
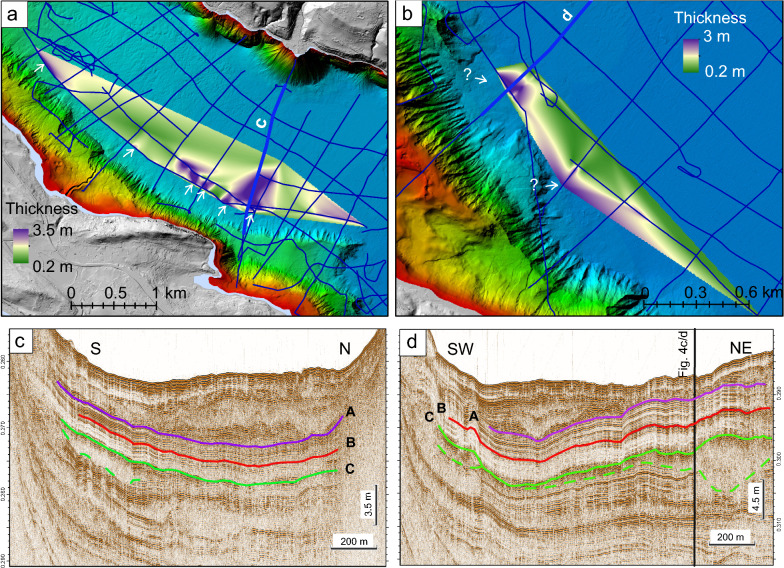
Thickness distribution maps of the smaller mass-movement deposits topped by Horizon C at the southern shore (**a** and **b**) and two pinger profiles showing the MMDs within the lacustrine sediment column (**c** and **d**). The outline of the map and the position of the pinger profiles are shown in Fig. 1a. The thicker blue lines in **a** and **b** mark the position of the pinger profiles in **c** and **d**

Figure 4 shows two high-resolution pinger reflection seismic profiles that are acquired with a 3.5 kHz pinger source. Profile a/b is recorded on a similar track as the profile in Fig. 3, profile c/d is oriented perpendicular to a/b and records the more distal part of the deposit outside of the amphitheater morphology. On these pinger profiles, only the upper ~ 20 m of the Holocene sedimentary record is imaged, characterised mainly by (1) low to high-amplitude, continuous and parallel reflections, interpreted as normal hemipelagic sedimentation of fine-grained particles intercalated with turbidites and by (2) chaotic to transparent seismic facies, interpreted as mass-movement deposits (MMDs). From top to bottom, three seismic horizons, A, B and C, are identified and can be traced over the entire deep basin. Therefore, these reflections represent useful time markers for the seismic stratigraphy. Horizon C describes the top of a transparent to chaotic seismic facies corresponding to the top of the large mass-movement deposit already described on the airgun profiles in Fig. 3. The base of this mass-movement deposit is not visualized on the pinger profiles due to limited seismic penetration. In southern direction, horizon C constitutes the top of a thin unit of transparent facies that fades out, as it can be seen on Fig. 4c/d. This part is the frontally emergent part of the mass movement (Moernaut et al., 2011) and is intepreted as a mass flow deposit (Schnellman et al., 2005). There is no evidence in the seismic data that a (mega-)turbidite was deposited on top of the mass movement deposits as it was observed in similar cases (e.g., Van Daele et al., 2013). Furthermore, horizon C represents the top of further smaller mass-movement deposits originating from the western (sublacustrine) slope (Fig. 5). We interprete these small deposits as several smaller mass movements as the thickness distribution map shows several depocentres. In addition, this western slope is characterised by failure scars and by MMDs at the toe of the slope, shown on the bathymetric map (Additional file 1: Fig. S2). Thus, some of these might be related to horizon C.

A second horizon B was defined in the pinger profiles (Fig. 4b, d). This high-amplitude horizon can be followed through the entire deep basin. No chaotic to transparent facies can be related to this horizon, thus it might be created by a 10–20 cm thick layer of material whose properties contrast to the upper and lower sediments, e.g., a sandy layer.

A third reflection, named horizon A (Fig. 4), represents the base of a sequence that has been defined by Wirth et al. (2011) previously. This sequence is characterized in the northwestern part by a chaotic to transparent seismic facies (Fig. 4). The base, horizon A, is equivalent to horizon b in Wirth et al. (2011) and interterpreted as the time marker of the Kander deviation into Lake Thun (Wirth et al., 2011). The chaotic facies within this sequence is interpreted as sediment reworked by numerous mass-movement episodes originating at the fast accumulating Kander Delta (Wirth et al., 2011).

**Fig. 6:**
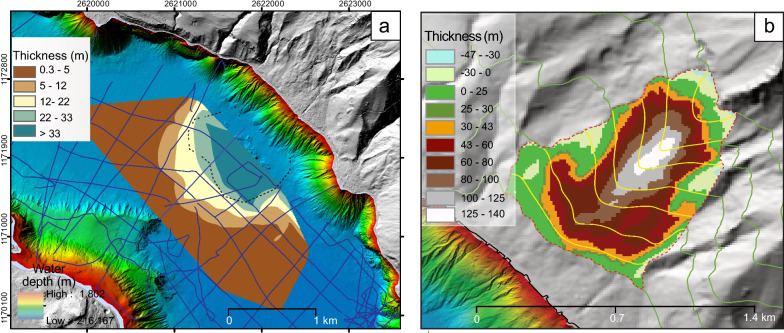
Off- and on-shore thickness maps. **a** Off-shore thickness map of the seismic facies related to the rockslide-induced deposit (with basin plain sediments). The limits of the amphitheatre structure are shown with a black dashed line. **b** On-shore thickness map of the potentially terrestrial failed mass as the difference between the present and reconstructed topography. Negative thickness values are due to extrapolation uncertainties. Source of the digital elevation model: Swisstopo, Federal Office of Topography

A thickness distribution map of the mass movement topped by horizon C can be generated combining airgun and pinger data (Fig. 6a). While both types of reflection seismic data allow imaging the top of the mass movement, only the airgun data visualizes the base of the deformed sediment volume (Fig. 3). The thickness map of the deposit and deformed lake sediments shows its largest thickness in the central part of the amphitheatre structure. Considering horizon C in Fig. 4 as top and the green dashed line in Fig. 3 as base of the deposit, a thickness of up to 100 m is calculated. As already stated above when interpreting the reflection seismic data, this thickness estimation includes also the basin plain sediments deformed by the impact of the falling rock mass. When considering only what is interpreted as deposit related to the moved mass mixed up with lacustrine sediments (delimited by the white dashed line in Fig. 3b), a thickness of ~ 30–40 m is calculated. The amphitheater structure delimits the deformation front of the underlying sediments and not the deposit. Outside the amphitheater structure the deposit thins rapidly out.

### Volume calculation of failed mass

As mentioned in the method chapter (3.5), the mass-movement volume estimation was done for the on-shore and off-shore traces of the event.

The on-shore pre-failure topography reconstruction (Fig. 6b) resulted in a total volume of ~ 30 × 10^6^ m^3^ (Additional file 1: Table S2). This result is affected by several physical (e.g., depicted failure area, location of the reference profile, scar position, amount of “accelerated post-failure erosion”) and computational (e.g., cell size, amount of profiles used for pre-failure surface reconstruction) factors influencing the on-shore pre-failure volume reconstruction. Changing the resolution of the reconstructed pre-failure topography by doubling the cell size from 10 to 20 m leads to a decrease in failure volume of 6% (Scenario Cell20), while an increase in resolution from 10 to 5 m shows a 3.1% increase in failed volume (Scenario Cell5). A reduction in applied profiles from 7 (P1–P7) to 4 (P1, P3, P4, P6) causes a 9% volume increase (smoother/averaged pre-failure topography). Furthermore, a freshly failed slope is likely more susceptible to erosion, leading to an overestimation of the volume when using todays topography for the calculation. Assuming an increase in denudation rate from an average 0.1 mm/year (Delunel et al., 2020, Haslital catchment-wide denudation rate) to 0.2 mm/year due to an initial high post-failure debris flow activity over the course of the last 2.4 kyrs, results in an overestimation of the failed volume when using today’s digital elevation model. We can take this into account by considering e.g., 1 m or 2 m of additionally eroded material (Eros1, Eros2), which leads to a calculated volume reduction with respect to the reference scenario of 1.9 or 3.7%, respectively. After testing various parameters, the onshore reconstructed failure volume (standard scenario) lies within an acceptable ± 10% error range.

The off-shore volume of deformed sediment and rock debris is estimated at around 60 × 10^6^ m^3^. This volume is calculated by multiplying the area affected by the “amphitheater” morphology of ~ 0.75 × 10^6^ m^2^ times by an average thickness of 80 m of deformed and deposited sediment and rock mass. This volume includes also the deformed basin plain sediments, since the bottom boundary of the rockslide deposits cannot be clearly defined in the reflection seismic data. This calculated off-shore volume is therefore overestimating the terrestrial failed rock mass. As stated above (Sect. 4.1), the average thickness attributed to the failed rock mass is estimated to around 30 m. Thus, we suggest a volume of ~ 20 × 10^6^ m^3^ for the mobilized rock mass and debris that deposited on the lake floor. However, this estimation is affected by large uncertainties as the reflection seismic data do not allow a clear distinction of the deformed basin plain and the failed rock masses. In addition to this volume deposited in the basin, there might be rock mass deposited on the slope. The slope gradient of ~ 40° at this location compared to 80° (Additional file 1: Fig. S1) at adjacent undisturbed slopes suggests that this difference in steepness might be related to rock-mass deposits.

Comparing the volumes of ~ 20 × 10^6^ m^3^ and 30 × 10^6^ m^3^, assessed from off-shore and on-shore datasets respectively, although the same order of magnitude, the terrestrial volume is larger than the volume estimated from the lake-sediment record. Reasons for this might be the denudation rates in the on-shore area that we did not take into account and, for the off-shore estimation, a certain volume of debris that was deposited on the slope. Thus, both volume estimations are affected by uncertainties as outlined above.

### Sediment facies

**Fig. 7:**
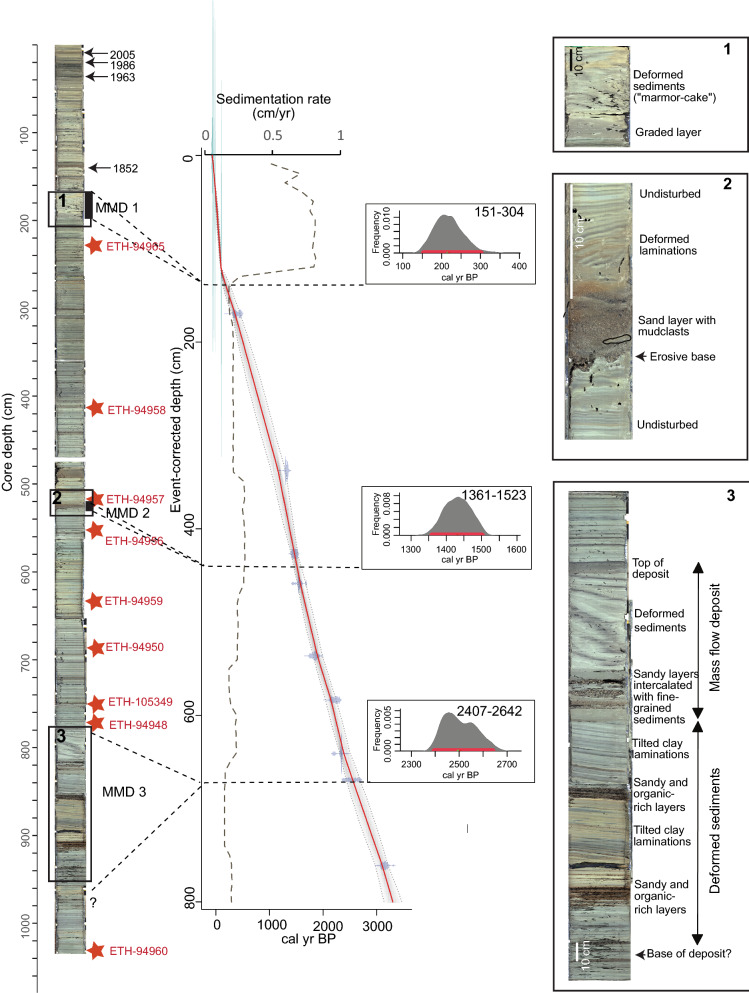
Sediment-core stratigraphy and age-depth model of core THU18-Ku-3. The sedimentological details of the MMDs are shown in zooms 1–3. The position of the radiocarbon ages is marked with a red star and the respective sample number (Table 1). The position of the historical time markers is highlighted with an arrow and the year. The age-depth model is shown in the middle part of the figure: with the calibrated ^14^C ages in blue, the grey pointed lines delimiting the grey envelope represent the 95% confidence interval of the ages while the red curve represents the “best” model based on the weighted mean age of each depth. The dashed grey curve represents the sedimentation rate in cm/year. The ^14^C age distribution of selected depths are also displayed. The red bars mark the 95% confidence interval. MMD: Mass-movement deposit

The sediments of core THU18-Ku-03 (Fig. 7) are characterised by light to dark grey-blueish fine-grained sediments (interpretated as hemipelagic sedimentation) intercalated by cm-scale normally graded layers that are interpreted as turbidites. The maximum grain size of these turbidites range between fine and coarse sand. 23 turbidites with thicknesses larger then 2 cm were identified and are listed in Additional file 1: Table S1 (similar to the turbidite facies observed in Sturm and Matter (1972). There is a general increase in the number of turbidites (10 of 23 turbidites) in the upper 2 m of the sediment core (Additional file 1: Table S1). The turbidites located at 6.6 cm and 151.9 cm depth are interpreted to be caused by the 2005 and 1852 flood events, through core-to-core correlation with dated sediment cores of Wirth et al. (2011) (correlation of short cores in Additional file 1: Fig. S1, Table S1) and are used as time markers for the age-depth model.

In addition to the turbidites, three units of deformed sediments are identified in core Ku-03:

1. At 2 m core depth, a 20 cm-thick layer occurs of deformed and chaotic, light brown to greyish fine-grained sediments (Fig. 7 zoom 1). The lower 6 cm consist of a fine-grained graded greyish sediments, they are covered by 14 cm of deformed sediments with a “marmor-cake” signature. This suggests a turbidite flow at the base and fluidised remobilised sediments on top. This unit is defined as a mass-movement deposit (MMD 1).
2. At 5.25 m, a 10 cm-thick layer is observed that consists of a brownish to dark greyish sandy 5 cm-thick layer with incorporated small mudclasts (2–3 mm thick) and an erosive base. This sandy layer is followed by a 5 cm-thick interval of deformed grey to dark grey mm-thick laminations (Fig. 7 zoom 2). This unit is named MMD 2.
3. Between 7.9 and 9.6 m core depth, the sediment can be described from bottom to top as follows: (1) the lower 1.2 m are composed of cm-thick, sandy, brownish and organic-rich layers alternating with mm-thick, fine grained, grey to dark grey and brownish, tilted laminations up to a depth of 8.40 m, follwed by (2) a 10 cm-thick unit of sandy layers within a muddy matrix, (3) a 40 cm-thick sequence of muddy, grey, deformed and tilted sediments ressembling a streched “marmor-cake” (Fig. 7 zoom 3). The top of this unit is characterised by a 3 cm-thick grey homogenous clayey layer with a mm-thick light grey cap. While the top of this unit seems well defined, the base is unclear. We define the base at the first appearance of the sandy organic-rich layers in combination with the tilted muddy layers (Fig. 7 zoom 3). The entire unit is interpreted as beeing related to a mass movement (MMD 3) where the lower part up to 8.40 m depth most probably consists of deformed lacustrine sediments while the upper part is most likely a mass-flow deposit (e.g., Schnellmann et al., 2005, 2006). As no phases of “calm” sedimentation can be observed within this unit, the deposit is the result of one single event.

### Seismic-to-core correlation

The seismic-to-core correlation is shown in Additional file 1: Fig. S4. The correlation was made after careful observation of the seismic reflection data and the sediment core. The three most prominent high-amplitude horizon (A, B and C) observed in the seismic data were matched to facies changes in the sediment core using a velocity of 1500 m/s for the conversion from time to depth of the seismic reflection data. Horizon A is correlated to the graded layer at the base of MMD 1, horizon B correspondes most probably to the sandy base of MMD 2 and horizon C is interpretated as the top of MMD 3.

### Age-depth model and event dating

The age-depth model covered by core THU18-Ku-03 is shown in Fig. 7, with the 95% confidence interval of the modeled ages and the red line indicating the weighted mean age of each depth. The age-depth model is based on the event-corrected depth. Based on this model, the sedimentation rate varies around 0.2 cm/year from 8 to 1.3 m core depth using the event-corrected depth. From 1.3 to 0.5 m the sedimentation rates jumps to ~ 0.8 cm/year and decreases again to ~ 0.4 cm/year for the uppermost 0.5 m. These sedimentation rates fit within the ranges proposed by Sturm and Matter (1972) and Wirth et al. (2011). The modeled ages of the 3 MMDs defined in the sediment core are as follows (with a 95% confidence interval, red bar in Fig. 7):

MMD 1 is dated at 304–151 cal year BP (1646–1799 cal AD).

MMD 2 is dated at 1523–1361 cal year BP (427–589 cal AD).

MMD 3 is dated at 2642–2407 cal year BP (690–440 cal BC).

This new age-depth model changes the previous interpretation of the sedimentation rate of Wirth et al. (2011) of Lake Thun. Wirth et al. (2011) interpreted horizon C (MMD 3) as being related to the event of 598/599 AD mentioned by Fredegarius (1888) in the absence of longer sediment cores. The new data, however, show that this reflection is about 1000 years older.

### Impulse wave assessment

For assessing the tsunamigenesis of such a subaerial mass movement, we investigated a scenario with the estimated volume of ~ 20 × 10^6^ m^3^ (calculated on-shore volume, scenario I). However, as material is potentially present on the lake slope we also considered an event with a larger volume of 35 × 10^6^ m^3^ (calculated off-shore volume plus added volume on lake slope, scenario II). Choosing these 2 different volumes was also done to show the effect of the volume on the wave amplitude. The governing parameters that were chosen as input values for the impulse wave assessment are given in Table 2. While most parameters directly result as individual values from either topographic and bathymetric data or the previously described observations, a range was chosen for the dynamic bed friction angle *δ* as governing parameter of the slide impact velocity $$ V_{s}$$ (Eq. 1). Consequently, $$ V_{s}$$ and all derived quantities are presented as value ranges in Table 2. For *δ* between 15 and 17°, $$ V_{s}$$ from 17 to 29 m/s result. Today’s still water depth *h* of approximatley 200 m in the vicinity of the slide impact location was increased by 7–8 m to account for ~ 2500 years of sedimentation.

**Table 2:** Summary of the input parameters and estimated wave characteristics of the tsunami assessment for scenario I and scenario II (in parantheses)

Parameter	Symbol	Value (per profile)				Unit
		A	B	C	D	
Drop height of the slide centroid	Δ*z*_*sc*_	242	242	242	242	m
Dynamic bed friction angle	*δ*	15…17	15…17	15…17	15…17	°
Slide impact velocity	$V_{s}$	17…29	17…29	17…29	17…29	m/s
Bulk slide volume	$\rlap{--} V_{s}$	20·10^6^ (35·10^6^)	20·10^6^ (35·10^6^)	20·10^6^ (35·10^6^)	20·10^6^ (35·10^6^)	m^3^
Slide thickness	*s*	45 (60)	45 (60)	45 (60)	45 (60)	m
Slide width	*b*	400 (500)	400 (500)	400 (500)	400 (500)	m
Bulk slide density	*ρ*_*s*_	1600	1600	1600	1600	kg/m^3^
Slide impact angle	*α*	18	18	18	18	°
Still water depth	*h*	210	210	210	210	m
Radial distance	*r*	3100	3600	8100	6400	m
Wave propagation angle	*γ*	0	12	68	− 78	°
Impact radius for γ = 0°	*r*_0,0°_	407…466 (456…521)	407…466 (456…521)	407…466 (456…521)	407…466 (456…521)	m
Initial first wave crest amplitude	*a*_0,*c*1_	30…39 (40…52)	30…39 (40…52)	30…39 (40…52)	30…39 (40…52)	m
Initial second wave crest amplitude	*a*_0,*c*2_	23…26 (26…29)	23…26 (26…29)	23…26 (26…29)	23…26 (26…29)	m
First wave crest amplitude	*a*_*c*1_	2…4 (4…6)	2…3 (3…5)	0.2…0.4 (0.4…0.7)	0.3…0.5 (0.5…0.8)	m
First wave trough amplitude	*a*_*t*1_	5…7 (7…11)	4…6 (5…8)	0.4…0.7 (0.7…1.2)	0.5…0.9 (0.8…1.4)	m
Second wave crest amplitude	*a*_*c*2_	11…13 (13…16)	10…12 (12…14)	4…5 (4…5)	3…4 (4…5)	m
Run-up angle	*β*	14	12	6	24	°
Run-up height (second wave)	*R*	34…40 (39…46)	31…37 (36…43)	13…16 (15…18)	9…11 (11…13)	m
Run-up height (second wave)	*R*_2_	54…68 (66…84)	50…63 (61…78)	21…26 (25…32)	12…15 (15…18)	m

Notation according to Evers et al. (2019) and Kastinger et al. (2020). Wave impact locations for propagation profiles A, B, C, D shown in Fig. 1

For scenario I, the maximum wave amplitudes within the lake were estimated at 30–39 m (scenario II: 40–52 m) above the still water level and occurred at the first wave crest *a*_0,*c*1_ at a distance *r*_0,0°_ between 407 and 466 m (scenario II: 456 and 521 m) from the impact shore. However, during propagation the first wave crest amplitude *a*_*c*1_ decays at a higher rate and the second wave crest amplitude *a*_*c*2_ therefore becomes the decisive wave characteristic for run-up estimations. With the run-up equation by Evers and Boes (2019) the maximum run-up heights were induced at the shore of Bürg hill (A on Fig. 1) with 34–40 m for scenario I and 39 and 46 m for scenario II. With increasing radial distance *r* and propagation angle *γ* smaller run-up heights result. At the location of today’s town of Thun (D on Fig. 1), run-up heights *R* between 9 and 11 m for scenario I and 11 and 13 m for scenario II were estimated. Applying the run-up equation by Kastinger et al. (2020) yields about 40–80% larger run-up heights *R*_2_ for the second wave crest.

## Discussion

The combination of reflection seismic profiles, the multibeam bathymetry and the sediment-core data allows reconstructing the sedimentation history of the past ~ 2500 years of Lake Thun. The sediments retrieved in sediment core THU18-Ku-03 are characterised by fine-grained background sedimentation intercalated with cm-scale turbidites and mass-movement deposits. The new sediment core allows the dating of the upper 11 m of sediments and mainly three prominent seismic horizons (A, B and C; Fig. 4) that can be used as time markers throughout the deep basin. With this new age-depth model, horizons B and C are much older than previously estimated by Wirth et al. (2011). This data also highlights the importance of long sediment cores with datable material to reconstruct the story told by the sediment archive.

The uppermost horizon (A), dated at 1646–1799 cal AD was already identified by Wirth et al. (2011) as being related to the deviation of the Kander River in 1714 AD into Lake Thun. The chaotic-transparent facies was attributed to enhanced mass-movement activity and consequent turbidite deposition originating from the fresh and instable sediments of the Kander Delta (Wirth et al., 2011). A potential further reason for a major mass movement could be the 1729 AD Frutigen earthquake with a magnitude Mw 5.6 (ECOS-09; Fäh et al., 2011). The earthquake shaking potentially combined with the instable slopes of the Kander Delta that accumulated within only years to a substantial structure.

The historic reports of the ‘boiling lake’ and ‘fished washed ashore’ in 598/599 AD (Fredegarius, 1888) can be potentially interpreted as the effect of an impulse wave event. MMD 2 dated at 427–589 cal AD may be related to this historical event, although the dated interval is slightly older. This deposit with a thick sandy base is at the origin of the high-amplitude horizon B in Fig. 4. However, no further traces of a mass-movement deposit is found in the reflection seismic data (i.e., transparent or chaotic facies associated with horizon B). Thus, if this deposit is related to a mass movement, it must have been a small event compared to the prehistorical one.

**Fig. 8:**
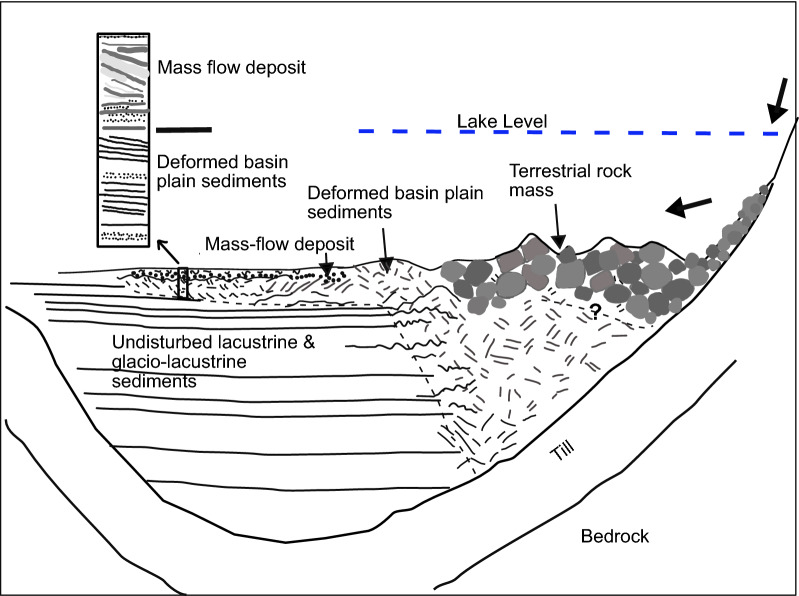
Schema of the off-shore deposition of the prehistorical terrestrial mass movement and the deformation of the basin plain sediments

MMD 3 is interpreted as being caused by a terrestrial mass movement. The 2642–2407 cal year BP old mass movement is characterised in the reflection seismic data by a thick deposit up to several tens of meters in thickness over an area of ~ 0.75 km^2^. The distal part of this deposit recorded in the sediment core consists of deformed basin plain sediments and a mass flow deposit. The main question that arises is whether the sedimentary deposit suggests a single event or a succession of several smaller events, a difference that is of major importance for the impulse wave modeling. As we do not have a clear signal of ‘calm’/regular lacustrine sedimentation within MMD 3, we suggest a single event. In addition, the amphitheatre visible on the multibeam map that shows simlarities with an impact crater is interpreted as an effect of the mass movement impact on the lake floor. Such an impact is rather expected from a large mass movement than from several smaller ones. Thus, the sedimentological and seismic facies, as well as the bathymatric map suggest a large prehistorical mass movement recorded in the seismic data and in the sediment core. Similar events were described in lakes and fjords (e.g., Lake Lucerne; Schnellmann et al., 2005; Hilbe et al., 2011, Aysen fjord; Van Daele et al., 2013). Making a comparison of MMD 3 with events described in Van Daele et al. (2013), we don’t have evidence for a clear basal shear surface responsible for ramping up of the deformation front (Fig. 8). Also we do not see evidence for any turbidite deposition within the amphitheatre area. Thus, the rock mass material is most likely present within the amphitheatre structure and on the adjacent slope where the rockmass source is located, indicated by the less steep slope angle in comparison to the undisturbed slope gradient of 80° (Additional file 1: Fig. S1). Outside of the amphitheatre deformed basin plain sediments can be found as in MMD 3 in core THU18-Ku-3.

If we consider as proposed a single event, with the above estimated volume of 20 and 35 Mio m^3^, impulse waves with a maximum wave crest amplitude between 30 and 50 m are estimated (Table 2). The impulse wave amplitude depends on the falling rock volume, the slide geometry and the dynamic bed friction angle governing the slide-impact velocity. However, compared to the slide impact velocity, the slide volume is of secondary importance for the wave generation. Thus, although the reconstructed volumes have uncertainties as their estimation is based on assumptions, the actually applied slide volume only plays a minor role in the impulse wave estimation.

**Fig. 9:**
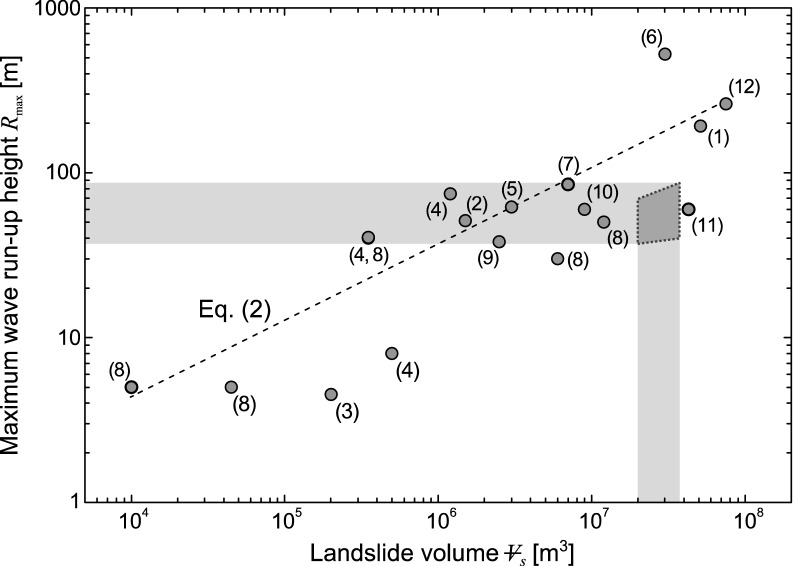
Maximum run-up height *R*_max_ versus landslide volume $\rlap{--} V_{s}$. Data are composed of Lake Thun mass movement (grey: *R*_max_ = 34…84 m, $\rlap{--} V_{s}$ = 20 × 10^6^ … 35 × 10^6^ m^3^; dotted) and historical cases as compiled by Clark et al. (2015) and Leithold et al. (2019) including (1) Haeussler et al., (2018), (2) Evans (1989), (3) Hancox et al., (2003), (4) Hansen et al., (2016), (5) Jørstad (1968), (6) Miller (1960), (7) Naranjo et al., (2009), (8) Norwegian Water Resources and Energy Directorate (2016), (9) Roberts et al., (2013), (10) Watt et al., (2009), (11) Wiles and Calkin (1992), (12) Zaniboni and Tinti (2014) (adapted from Leithold et al., 2019)

Figure 9 adapted from Leithold et al. (2019) shows the maximum measured run-up heights versus landslide volumes for historical impulse wave events. Leithold et al. (2019) derived an empirical prediction equation for the maximum run-up height with a power law regression. The prehistorical Lake Thun mass movement would have generated maximum run-up heights between 34 m (lower estimation of *R*) and 84 m at Bürg hill (upper estimation for *R*_2_) on the opposite shore of the slide impact location (Fig. 1, point A). Figure 9 shows that these maximum run-up heights *R*_max_ estimated with the computational procedures of Evers et al. (2019) and Kastinger et al. (2020) are within the scatter range of the historical events described by Leithold et al. (2019) with their prediction equation

2 $$R_{\max } = \, 0.063 \,\rlap{--} V_{s}^{0.4624}$$

which is given with a coefficient of determination *R*^2^ = 0.44. However, note that Eq. (2) neither accounts for the slide impact velocity (most important factor) and geometry, nor the radial distance and wave propagation angle from the slide impact location to the run-up slope. Consequently, the run-up heights of historical events shown in Fig. 9 scatter up to approximately one order of magnitude for similar landslide volumes. This may be demonstrated with a comparison to the Lituya Bay event in 1958 (6, Fig. 9): While the slide volume of 30.6 × 10^6^ m^3^ was within the estimated scenarios of the Ralligen slide, the wave run-up height of 524 m was approximately one order of magnitude larger (Slingerland & Voight, 1979). This deviation may be attributed to a much larger drop height at a steeper slope and consequently higher impact velocity of the Lituya Bay slide—estimated between 56 and 92 m/s (Slingerland & Voight, 1979; Heller, 2008)—as well as the shorter wave propagation distance of approximately 1300 m (Miller, 1960).

The induced impulse wave might not have affected habitants around Lake Thun. The time between 2642 and 2407 cal year BP (690–440 cal BC) corresponds to the Early Iron Age. During this age, the classical villages of pile dwellers around the Alps did not exist anymore. People were living rather away from lakes, and remnants of this time are not well preserved (written communication with Lukas Schärer from the archeological service of canton Bern). Around Lake Thun, only remnants of a potential grave and a pit have been found in Thun dated at 8th to fifth century BC and 8th to sixth century BC, respectively (Gubler et al., 2017; Othenin-Girard, 2018; Schärer & Ramstein, 2017), suggesting that the shores of Lake Thun were not inhabited at the time of the prehistorical mass movement.

The potential triggers of mass movements are diverse: heavy rainfall, erosion, temperature changes or extrem stresses such as earthquakes can be responsible for such events. Deciphering between these triggers, especially in prehistorical studies is a challenge and cannot be conclusive without additional evidence.

The occurrence of coeval mass movement deposits within one basin or several basins is considered as an evidence for a earthquake trigger. MMD 3 coincides with several smaller MMDs orgininating from the opposite shore (Fig. 5). Thus, this could point to a seismic trigger for the prehistorical MMD. However, the smaller MMDs might also be caused by the mass-movement-induced impulse wave.

The timing of the Lake Thun prehistorical mass movement potentially coincides with a rock avalanche that represents the base of the sedimentary sequence of Lake Oeschinen around 24 km South of Lake Thun and dated to 2379–2713 cal year BP by radiocarbon dating of wood pieces trapped in the sediments (Knapp et al., 2019) and to 2300 ± 200 year BP by ^36^Cl surface exposure dating of deposited boulders (Köpfli et al., 2018). The source volume of the Oeschinen rock avalanche is 37 × 10^6^ m^3^ (Köpfli et al., 2018) and has been discussed by Knapp et al. (2019) to be potentially earthquake-triggered. Considering the overlap in time between the Oeschinen rock avalanche and the Lake Thun rockslide and the distance between both sites, both mass movements can potentially be related to the same event. This timing is preciding a phase of potentially enhanced mass-movement activity (~ 2200 cal year BP) recorded in Swiss lakes (Kremer et al., 2017).

Another trigger for the rock failure could be wet and cool climatic conditions that were predominant during this period (Czymzik et al., 2013; Steinhilber et al., 2009; Trouet et al., 2009; Wanner et al., 2011; Wirth et al., 2013). These conditions were probably due to two lows in solar activity at ~ 2800 cal year BP and ~ 2500 cal year BP coming along with a preliminary negative situation of the North Atlantic Oscillation (Steinhilber et al., 2009; Trouet et al., 2009). The Monte Peron rock avalanche in the Dolomites (Italy) has also recently been dated at 2890 cal B and as trigger wet climatic conditions were discussed (Zolitschka et al., 2021). Heavy rainfall events or intense snow melt could have led to an incrased water infiltration, swelling of the marly components in the limestones and thus to an activation of one or several slide planes within the rock package.

We consider both earthquake-induced shaking as well as wet climatic conditions as potential triggering mechanisms for the prehistorical mass movement at Lake Thun. This event and also the Oeschinen event fall into a time period where the radiocarbon calibration curve is characterized by a plateau (Hallstatt plateau). This makes refining the dating for a better comparison of the ages of the events using radiocarbon challenging.

New evidence from the area of the failure scar of the prehistorical Ralligen mass movement shows that the area seems unstable (written communication with Stefan Strasky from Swisstopo, Swiss Federal Office of Topography). This asks for further investigations on the shore of Lake Thun for assessing the hazard related to mass movements and mass-movement-induced impulse waves.

## Conclusion

In this study we reconstructed a prehistorical mass movement in the area of Lake Thun using reflection seismic data and a 11 m-long sediment core. The prehistorical mass movement was dated at 2740–2440 cal year BP (790–490 cal BC, Early Iron Age) and is thus, much older than previously estimated. Thus, this study shows the importance of long sediment cores with datable material for the reconstruction of past events through lacustrine sediments. This mass movement represents with its on-shore volume of ~ 20 × 10^6^ m^3^ one of the larger events whose deposits have been found in Swiss lakes. The volume range as well as the timing is similar as for the Oeschinen rock avalanche that occurred 24 km away from Lake Thun in the Alps (Knapp et al., 2018; Köpfli et al., 2018). Considering the failed volume sliding as a single mass, as suggested by the impact crater structure on the lake floor, an initial wave amplitude between 30 and 50 m was estimated. In today’s city of Thun at the outflow of the Lake Thun a run-up height of approximately 10–30 m was modeled. Within the Early Iron Age this event might not have been noticed by the people as there is scarce archeological evidence of human presence around the lake at that time. Today, considering the densely populated area and the attractivity of Alpine lakes for tourism, the hazard of mass movements and the potentially cascading effect of impulse wave needs to be further assessed, especially in a context of changing climate. Enhanced rainfall in winter and/or melting permafrost in high-alpine regions increases the hazard of mass movements in the Alpine region.

## Supplementary Information

**Additional file 1.** Supplementary information comprising 4 Figures (Fig. S1–S4) and 2 Tables (Table S1–S2).

## Data Availability

The datasets used and/or analyzed during the current study are available from the corresponding author on reasonable request.
