# Supplementary material for: Traces of a prehistoric and potentially tsunamigenic mass movement in the sediments of Lake Thun (Switzerland)
Source: Swiss J Geosci. 2022 Apr 9;115(1):13. doi: 10.1186/s00015-022-00405-0 (PMC8994746; doi:10.1186/s00015-022-00405-0)
Supplement: Supplementary file 1 — Additional file 1. Supplementary information comprising 4 Figures (Fig. S1–S4) and 2 Tables (Table S1–S2). [file 15_2022_405_MOESM1_ESM.docx]

Traces of a prehistoric and potentially tsunamigenic mass movement in the lacustrine sediments of Lake Thun (Switzerland)

Katrina Kremer*, Stefano C. Fabbri**, Frederic M. Evers***, Nora Schweizer*, Stefanie B. Wirth****,*****

* Swiss Seismological Service, ETH Zürich, Sonneggstrasse 5, CH-8092 Zurich, Switzerland

** Insitute of Geological Sciences and Oeschger Centre for Climate Change Research, Baltzerstrasse 1+3, University of Bern, CH-3012 Bern, Switzerland

***Laboratory of Hydraulics, Hydrology and Glaciology (VAW), ETH Zurich, CH-8093 Zürich, Switzerland

**** Centre for Hydrogeology and Geothermics (CHYN), University of Neuchatel, Emile-Argand 11, CH-2000 Neuchatel, Switzerland

***** GEOTEST AG, Bernstrasse 165, CH-3052 Zollikofen, Switzerland

Supplementary Information:


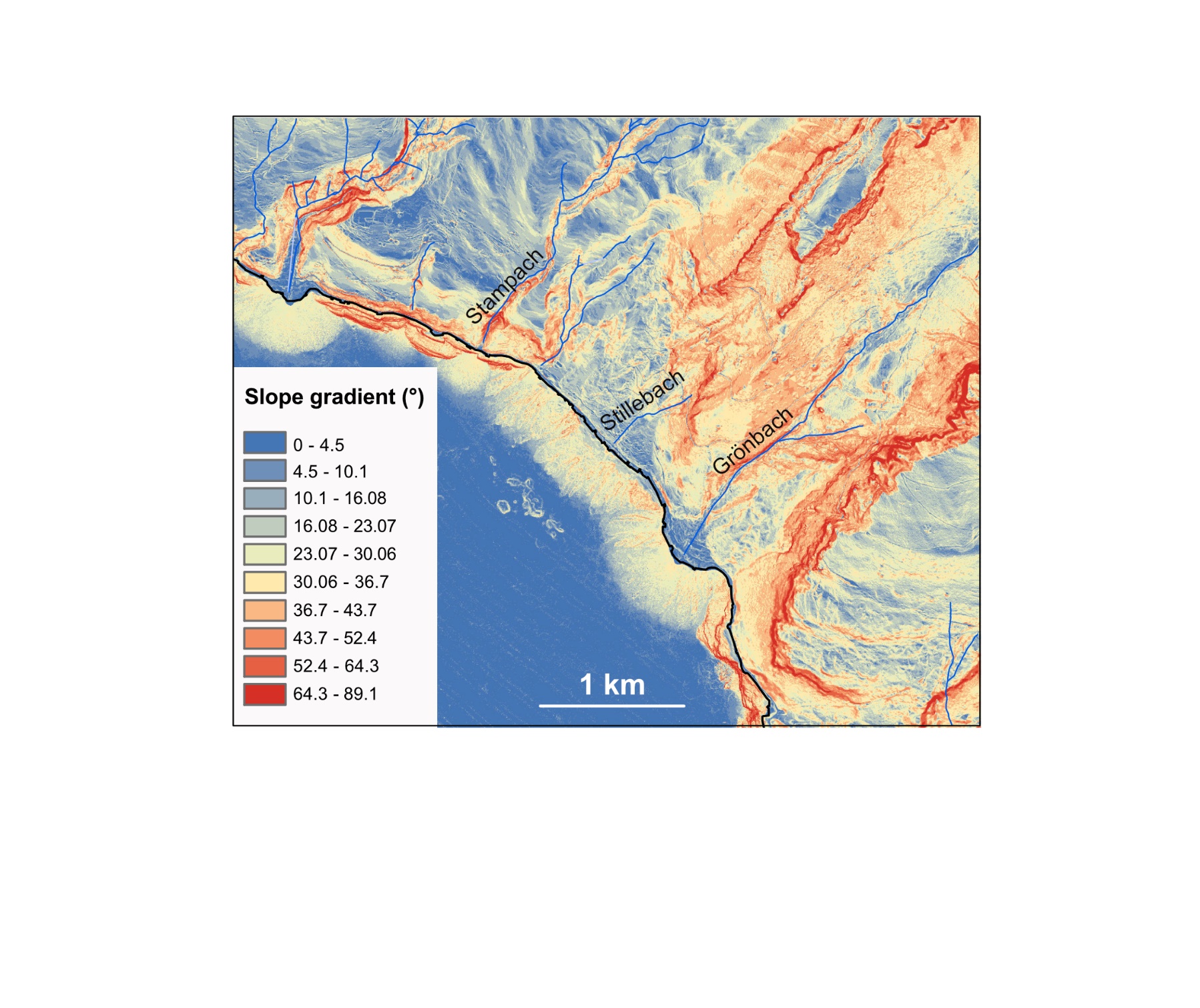


Fig. S1: Map of the slope gradient.


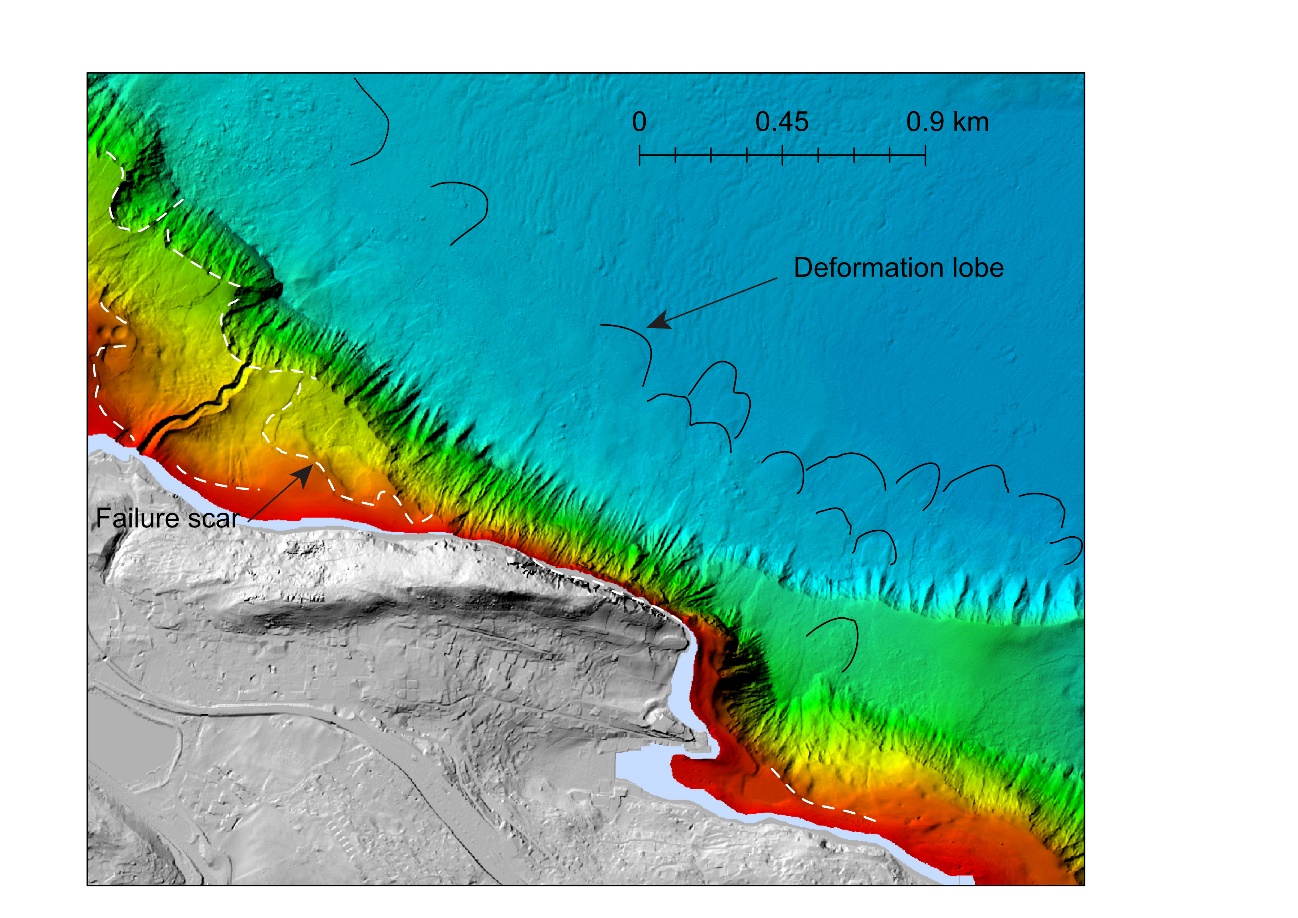


Fig. S2: A zoom on the southwestern submerged slope (region of the town of Spiez) where the bathymetric map is characterized by the occurrence of mass-movements deposits. The continuous black lines delimit the deformation lobes of the mass movements while the dashed white lines show potential failure scars.


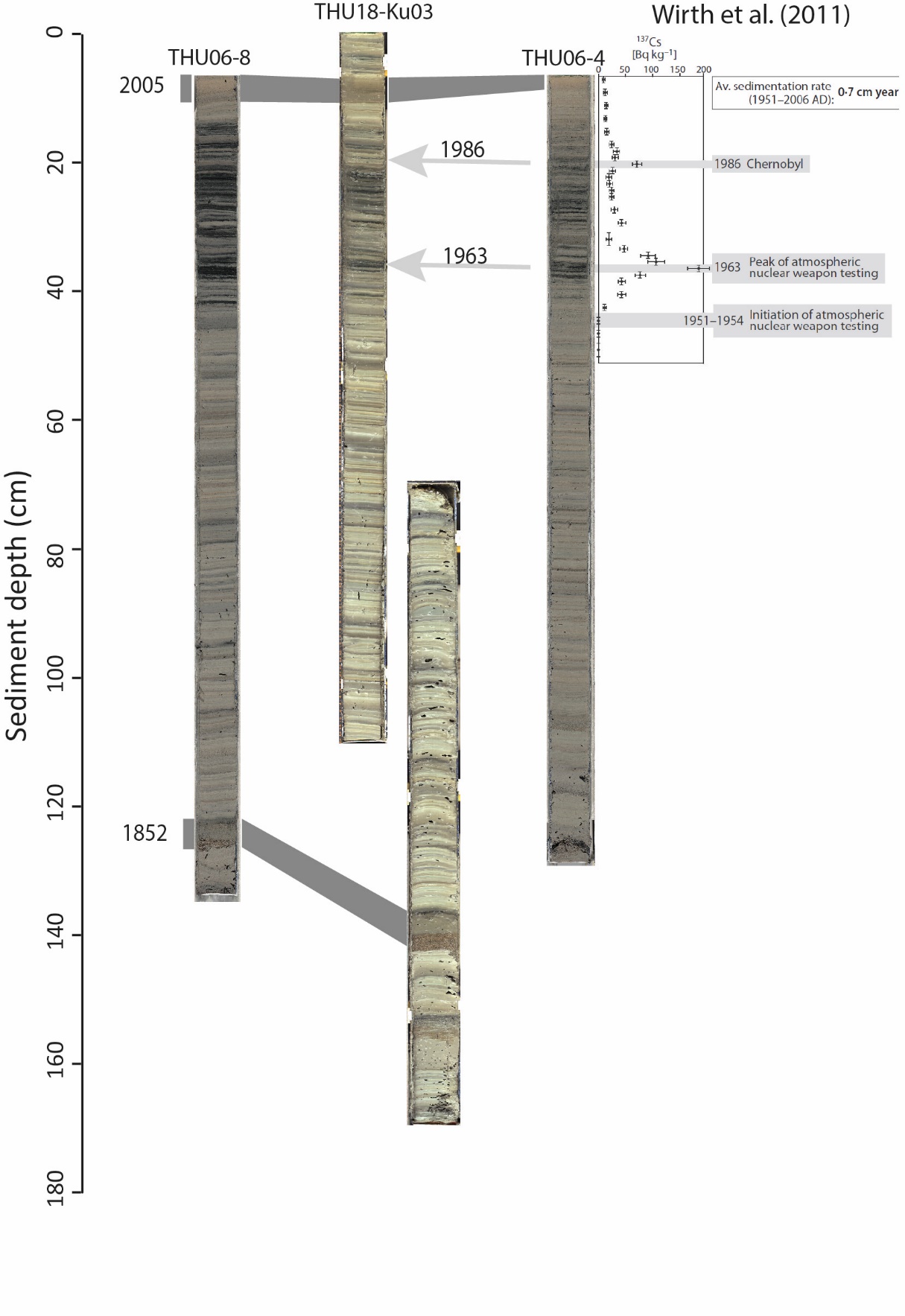


Figure S3: Core-to-core correlation. Correlation is done between previously dated sediment cores THU06-8 and THU06-4 of Wirth et al. (2011) and THU18-Ku-03 of this study. The core correlation shows the positions of the ^137^Cs peaks (1986 Chernobyl nuclear accident and 1963 peak of nuclear weapon testing) and the two historical floods of 2005 and 1852. These time markers were projected to THU18-Ku-03 to build the age model.


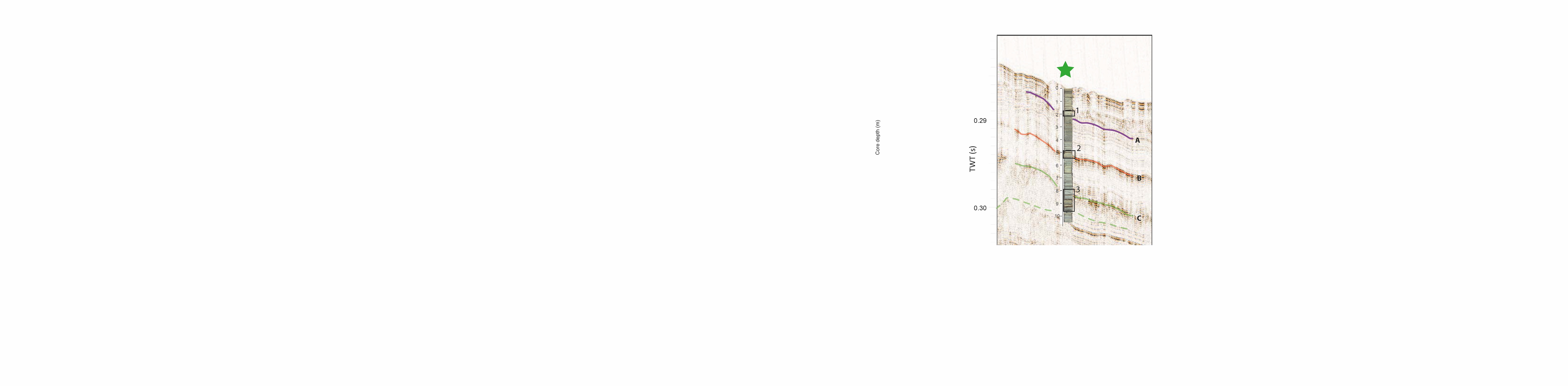


Fig S4: Seismic-to-core correlation. High-amplitude seismic horizons A, B and C correspond to the MMDs (mass-movement deposits) 1, 2 and 3, respectively. MMD 1 corresponds to the mass movement related to the Kander incision into Lake Thun. MMD 2 potentially corresponds to the historical 598/599 AD event. Horizon B highlights the sandy base of this deposit (Fig. 5C). MMD 3 corresponds to the prehistorical mass movement. Horizon C represents the top of this deposit. These reflections can be followed over the entire basin and can be used as time horizons for future studies.

Table S1: Positions of event deposits > 2 cm within individual core sections and the calculation of the composite depth without event deposits (ED).

| core section | Top of ED (cm) | Base of ED (cm) | Thickness of ED (mm) | Cumulative  thickness (mm) | Composite top of ED (cm) | Composite base of ED | Composite Depth without EDs |
| --- | --- | --- | --- | --- | --- | --- | --- |
| SC | 11.2 | 13.5 | 23 | 23 | 6.6 | 8.9 | 2.7 |
|  | 66.2 | 69.1 | 29 | 52 | 61.6 | 64.5 | 59.3 |
| C | 26.5 | 29.6 | 31 | 83 | 93.9 | 97 | 88.7 |
|  | 47.2 | 49.2 | 20 | 103 | 114.6 | 116.6 | 106.3 |
|  | 51.4 | 53.9 | 25 | 128 | 118.8 | 121.3 | 108.5 |
|  | 66.8 | 73.9 | 71 | 199 | 134.2 | 141.3 | 121.4 |
|  | 75.5 | 77.8 | 23 | 222 | 142.9 | 145.2 | 123 |
|  | 84.5 | 92.4 | 79 | 301 | 151.9 | 159.8 | 129.7 |
|  | 97 | 99 | 20 | 321 | 164.4 | 166.4 | 134.3 |
| E | 2.1 | 33.2 | 311 | 632 | 167.8 | 198.9 | 145.6 |
|  | 55 | 57 | 20 | 652 | 220.7 | 222.7 | 190.6 |
|  | 81.1 | 83.2 | 21 | 673 | 246.8 | 248.9 | 214.7 |
| F | 49.3 | 54.4 | 51 | 724 | 310.3 | 315.4 | 247.1 |
| G | 51 | 54.2 | 32 | 756 | 412.5 | 415.7 | 340.1 |
|  | 81.6 | 84.6 | 30 | 786 | 443.1 | 446.1 | 367.5 |
| I | 18.1 | 31.5 | 134 | 920 | 481.9 | 495.3 | 403.3 |
|  | 45.7 | 48.2 | 25 | 945 | 509.5 | 512 | 417.5 |
|  | 56.8 | 66.8 | 100 | 1045 | 520.6 | 530.6 | 426.1 |
|  | 93.1 | 95.2 | 21 | 1066 | 556.9 | 559 | 452.4 |
| J | 16.4 | 19.4 | 30 | 1096 | 573.3 | 649.7 | 466.7 |
|  | 90.2 | 92.8 | 26 | 1122 | 647.1 | 724.2 | 537.5 |
| K | 67.6 | 71 | 34 | 1156 | 720.8 | 853.5 | 608.6 |
| L | 23.6 | 100.3 | 767 | 1923 | 776.8 | 853.5 | 661.2 |
| M | 5.4 | 112.4 | 1070 | 2993 | 853.5 | 960.5 | 661.2 |
| N | 6.4 | 16 | 96 | 3089 | 960.5 | 970.1 | 661.2 |

Table S2: Onshore volume calculation sensitivity analysis, testing different parameters and their impact on the failed volume reconstruction. The “Standard” (Ref.) scenario considers no temporarily accelerated erosion due to high post-failure debris flow activity, and uses a standard cell size of 10 m for the pre-failure volume calculation onshore. Scenarios “Eros1” and “Eros2” consider temporarily accelerated erosion due to high post-failure debris flow activity. These scenarios suggest that the “Standard” model overestimates the failed volume, which is remedied by subtracting 1 m (Eros1) or 2 m (Eros2) from the vertical component of the failed volume. Scenario “Cell 5” and “Cell 20” evaluate the impact of using different cell sizes compared to the “Standard” scenario. Scenario Prof1346 evaluates the impact of using a reduced amount of profiles (only P1, P3, P4, P6 instead of P1-P7) for volume reconstruction.

| **Scenario** | **Profiles used** | **Cell Size**  **[m]** | **Accelerated Erosion due to high post-failure debris flow activity** | **Volume**  **[m^3^]** | **Change** | **Rounded**  **Change [%]** |
| --- | --- | --- | --- | --- | --- | --- |
| Standard (ref.) | P1 to P7 | 10 | 0 m | 29’859’516.9 | 0 | 0 |
| Eros1 | P1 to P7 | 10 | 1 m | 29‘302‘959.2 | 0.98136 | -1.9 |
| Eros2 | P1 to P7 | 10 | 2 m | 28‘750‘589.1 | 0.96286 | -3.7 |
| Cell5 | P1 to P7 | 5 | 0 m | 30‘781‘250.06 | 1.030868 | 3.1 |
| Cell20 | P1 to P7 | 20 | 0 m | 28‘077‘751.54 | 0.940328 | -6.0 |
| Prof1346 | P1, P3, P4, P6 | 10 | 0 m | 32‘533‘620.77 | 1.089556 | 9.0 |
